# Current and Emerging Treatment Paradigms in Colorectal Cancer: Integrating Hallmarks of Cancer

**DOI:** 10.3390/ijms25136967

**Published:** 2024-06-26

**Authors:** Clara Salva de Torres, Iosune Baraibar, Nadia Saoudi González, Javier Ros, Francesc Salva, Marta Rodríguez-Castells, Adriana Alcaraz, Ariadna García, Josep Tabernero, Elena Élez

**Affiliations:** 1 Vall d’Hebron University Hospital, E-08035 Barcelona, Spain; clara.salva@vallhebron.cat; 2 Medical Oncology Department, Vall d’Hebron University Hospital, Vall d’Hebron Institute of Oncology (VHIO), E-08035 Barcelona, Spain; ibaraibar@vhio.net (I.B.); nsaoudi@vhio.net (N.S.G.); fjros@vhio.net (J.R.); fsalva@vhio.net (F.S.); martarodriguez@vhio.net (M.R.-C.), jtabernero@vhio.net (J.T.); 3 Vall d’Hebron Institute of Oncology (VHIO), E-08035 Barcelona, Spain; adrianaalcaraz@vhio.net (A.A.); agarcia@vhio.net (A.G.)

**Keywords:** colorectal cancer, hallmarks of cancer, drug development, cancer biology, targeted therapies, immune checkpoint inhibitors

## Abstract

The treatment of unresectable metastatic colorectal cancer has evolved over the last two decades, as knowledge of cancer biology has broadened and new targets have emerged. ‘The Hallmarks of Cancer’ illustrate the crucial capabilities acquired by cells to become malignant and represent the evolution of knowledge of tumor biology. This review integrates these novel targets and therapies into selected hallmarks: sustaining proliferative signaling, inducing vasculature, avoiding immune destruction, genome instability and mutation, reprogramming cellular metabolism, and resisting cell death. The different strategies and combinations under study are based on treatments with anti-EGFR, anti-VEGF, and anti-HER2 agents, KRAS G12C inhibitors, BRAF and MEK inhibitors, and immune checkpoint inhibitors. However, new approaches are emerging, including vaccines, WEE1 inhibitors, and PARP inhibitors, among others. The further deciphering of cancer biology will unravel new targets, develop novel therapies, and improve patients’ outcomes.

## 1. Introduction

**Figure 1:**
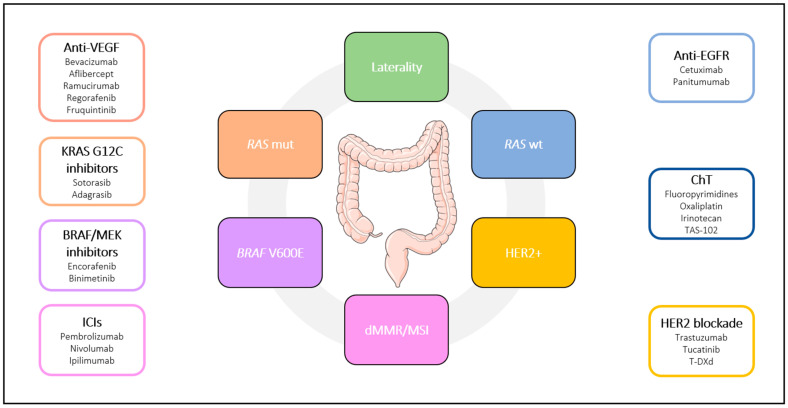
Current landscape of metastatic colorectal cancer (CRC) treatment. Abbreviations: VEGF, vascular endothelial growth factor; EGFR, epidermal growth factor receptor; dMMR, mismatch repair-deficient; MSI, microsatellite instability; ICIs, immune checkpoint inhibitors; mut, mutant; wt, wild-type; ChT, chemotherapy; TAS-102, trifluridine/tipiracil; T-DXd, trastuzumab deruxtecan.

Colorectal cancer (CRC) ranks as the third most prevalent cancer globally and stands as the second leading cause of cancer-related mortality [1]. The reduction in mortality rates in recent years can be attributed to enhanced screening programs, combined with improved treatment modalities [2]. The management of unresectable metastatic CRC has significantly evolved over the last two decades, and, in the contemporary landscape of precision oncology, therapeutic decisions increasingly hinge on biomarkers. Currently, testing for mutations in *KRAS*, *NRAS*, and *BRAF*, alongside evaluation for mismatch repair-deficient (dMMR) and microsatellite instability (MSI), constitute a fundamental component of the diagnostic process for establishing the treatment approach in metastatic CRC [3]. The identification of eventual *HER2* amplification is also recommended. Nevertheless, tumor responses to corresponding targeted therapies often fall short of expectations; this may be attributed to the inherent tumor heterogeneity and genetic complexity of CRC [4], as well as to the appropriateness of biomarkers and patient selection criteria. Notwithstanding these challenges, advances in the treatment of metastatic CRC have been notable in recent years, leading to a substantial improvement in overall survival (OS) figures, and consequently, various therapeutic options are now available in routine clinical practice (Figure 1).

Advances in cancer therapy have gone hand in hand with the broadening of our body of knowledge of cancer biology. Cancer cells possess the ability to continuously proliferate, invade other tissues, and avoid cell death. A description by Hanahan and Weinberg in 2000 of ‘The Hallmarks of Cancer’ was intended to provide a unified explanation for the crucial functional capabilities that human cells acquire when transitioning from the state of normalcy to neoplastic growth, identifying the set of characteristics that define malignancy. Initially, six aspects were described: sustaining proliferative signaling, evading growth suppressors, evading apoptosis, unlimited replicative potential, sustained angiogenesis and tissue invasion, and metastasis [5]. Subsequently, two more aspects were added: reprogramming cellular metabolism and avoiding immune destruction. Moreover, genome instability and tumor-promoting inflammation were proposed as means by which tumor cells could acquire these traits, termed “enabling characteristics” [6]. The most recent update incorporates novel parameters, including phenotypic plasticity, non-mutational epigenetic reprogramming, a polymorphic microbiome, and senescent cells [7].

The improvements in our comprehension of tumor biology are reflected in the diversity of new drugs being tested in clinical trials, as new molecular targets are brought to light. This review aims to integrate these novel targets and therapies within selected aspects of the framework of the hallmarks of cancer, in order to delve into the complex, intricate scenery of advanced CRC treatment and drug development.

## 2. The Hallmarks of Colorectal Cancer

### 2.1. Sustaining Proliferative Signaling

**Figure 2:**
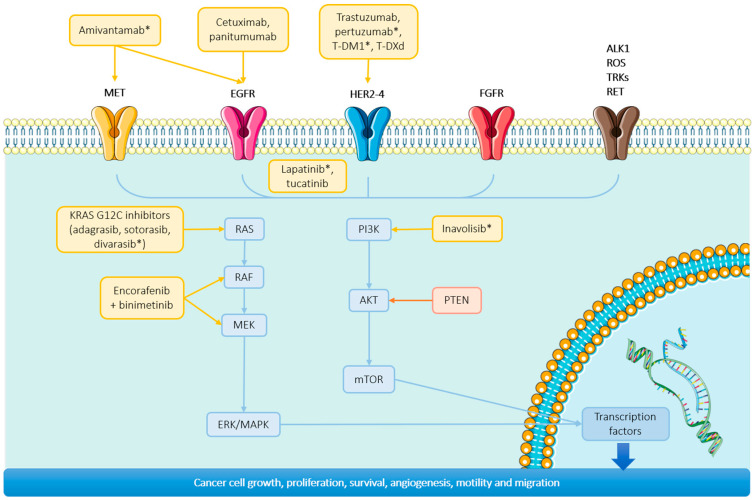
Targeted therapies in RAS-RAF-MAPK pathway. Approved treatments and treatments under investigation in RAS-RAF-MEK-ERK and PI3K-AKT-mTOR pathways. Treatments in ongoing clinical trials are marked with an asterisk (not approved by the FDA). Abbreviations: T-DM1, trastuzumab emtansine; T-DXd, trastuzumab deruxtecan.

In CRC, proliferation is sustained through the epidermal growth factor receptor (EGFR), also known as HER1, as well as through HER2/ERBB2, HER3/ERBB3, and HER4/ERBB4. These four receptors constitute a family of transmembrane receptors within the broader group of receptor tyrosine kinases (RTKs). Their dimerization activates different signaling cascades, including RAS-RAF-MEK-ERK, PI3K-AKT-mTOR, and JAK-STAT, which lead to cell growth, proliferation, angiogenesis, and survival (Figure 2) [8].

#### 2.1.1. EGFR

Anti-EGFR antibodies (cetuximab and panitumumab) have improved outcomes in patients with *RAS* wild-type advanced CRC [9,10], whereas activating mutations in *KRAS* or *NRAS*, present in 52% of metastatic CRC [11], provide resistance to anti-EGFR therapies, as the downstream activation of the signaling pathway is independent of EGFR. Similarly, mutations in *BRAF* and *PIK3CA* diminish the response to these antibodies.

EGFR and MET dimerize to promote oncogenic signaling, and in non-small-cell lung cancer (NSCLC), these receptors can compensate for one another when inhibited individually [12]. Amivantamab is a fully human bispecific antibody targeting EGFR and MET that has shown clinical activity against tumors with activating *EGFR* mutations, *EGFR* resistance mutations, or MET pathway activation. It was first approved for locally advanced or metastatic NSCLC with *EGFR* exon 20 insertion mutations, based on results from the CHRYSALIS trial [13]. In advanced CRC, amivantamab is being tested in the OrigAMI-1 phase Ib/II trial (NCT05379595) in monotherapy and in addition to standard-of-care chemotherapy (5-fluorouracil, folinic acid, and oxaliplatin [FOLFOX] or 5-fluorouracil, folinic acid, and irinotecan [FOLFIRI]).

#### 2.1.2. HER2

*HER2* alterations are present in only 1–4% of patients with CRC, with an enrichment in left-sided tumors and tumors located in the rectum, and it confers a poor prognosis [14,15]. Trastuzumab, one of the first drugs used to target HER2, binds to the extracellular domain of the receptor, blocking normal functioning. Nevertheless, resistance mechanisms to HER2-targeted therapy have been uncovered, such as mutations in *HER2* that modify the protein’s structure, the overexpression of other RTKs, or the internalization of the receptor, thereby diminishing its presence on the cell membrane surface [16]. A combination of trastuzumab with lapatinib, an EGFR and HER2 tyrosine kinase inhibitor (TKI), has shown an improved response in previously treated *RAS* wild-type, HER2-positive metastatic CRC patients in the phase II trial HERACLES-A, with an overall response rate (ORR) of 30%, a median progression-free survival (PFS) of 9.5 months, and a median OS of 11.5 months [17]. Additionally, dual therapy with trastuzumab and pertuzumab, also an inhibitor of the extracellular domain of HER2, gave an ORR of 32% in *HER2*-amplified metastatic CRC, with a potential OS benefit [18].

Trastuzumab deruxtecan (T-DXd) is an antibody–drug conjugate (ADC), meaning that, not only does it bind to HER2, blocking signaling, but furthermore, once bound, the receptor is internalized along with deruxtecan, a topoisomerase I inhibitor, causing structural damage to the DNA, leading to cell death. ADCs have revolutionized breast cancer treatment and are under investigation in other HER2-positive tumors. In CRC, T-DXd has demonstrated activity in patients with HER2-expressing refractory metastatic CRC in the phase II trial DESTINY-CRC01, with an ORR of 45% and a disease control rate (DCR) of 83% [19].

Tucatinib is an oral selective TKI against HER2 that was combined with trastuzumab in patients with *RAS* wild-type CRC harboring an amplification or overexpression of HER2 in the MOUNTAINEER trial, resulting in an ORR of 55%, median PFS of 6.5 months, and median OS of 17.3 months [20]. A clinical trial of its combination with mFOLFOX6 for frontline HER-positive metastatic CRC is currently recruiting (MOUNTAINEER-03, NCT05253651).

#### 2.1.3. KRAS

The activation of EGFR and other RTKs triggers several signaling pathways, with RAS-RAF-MEK-ERK being one of the most important. The phosphorylation of RAS starts a chain reaction that activates RAF, MEK, and ERK, which in turn translocate into the nucleus, regulating multiple transcription factors and gene expressions, controlling growth, proliferation, differentiation, and migration. This pathway also has the ability to activate PI3K and the downstream signaling pathway [8].

*KRAS*, *NRAS*, and *HRAS* belong to the *RAS* oncogene family, one of the most frequently mutated gene families across all cancers, with nearly half of all CRC patients presenting with *RAS* mutations [21]. With this pathway independent of upstream activation, these mutations are associated with a poor prognosis and resistance to anti-EGFR therapies. Over the last two decades, different strategies have been used to target these oncogenes without success, until the recent development of KRAS G12C inhibitors.

The *KRAS G12C* mutation is found in approximately 4% of patients with CRC and confers a particular tridimensional shape to the protein, with a binding pocket called the switch-II pocket [22]. This characteristic has allowed the development of various compounds that covalently bind to the pocket, maintaining the protein in its inactive form. Sotorasib (AMG 510) showed activity in solid tumors harboring the *KRAS G12C* mutation, including CRC. The phase I/II CodeBreaK100 trial reported a 74% DCR and an ORR of 7% in patients with CRC, with 12% grade 3 or 4 events and no dose-limiting toxicities [23]. It is also being studied in combination with other therapies. Studies with preclinical models showed that single-agent KRAS G12C inhibition in this CRC context produced an immediate upregulation of EGFR, leading to the development of dual-blockade therapeutic strategies with the addition of an EGFR-directed monoclonal antibody [24]. Recently, results from the CodeBreak300 trial have been published, comparing the combination of sotorasib and panitumumab versus (vs.) standard-of-care in chemorefractory metastatic *KRAS G12C*-mutated CRC, showing an improvement in the median PFS (5.6 months vs. 2.2 months) and in ORR (26.4% vs. 0%) [25]. Adagrasib (MRTX849) is another KRAS G12C inhibitor that has shown activity in previously treated NSCLC and CRC patients in the phase I/Ib KRYSTAL-1 study [26] and is being studied in combination with cetuximab. Results from the phase I/II trial demonstrated an ORR of 46% and median PFS of 6.9 months with combination therapy [27]; this combination is being evaluated in the ongoing phase III KRYSTAL-10 study in a second-line setting (NCT04793958). A third KRAS G12C inhibitor, divarasib, is under investigation as a combination therapy with cetuximab in an ongoing phase Ib trial. Preliminary data reported an ORR of 62.5% and a median PFS of 8.1 months in 24 patients with *KRAS G12C*-mutated CRC [28].

#### 2.1.4. BRAF

Further down in the pathway is the Raf protein family, including C-RAF/RAF-1, B-RAF and A-RAF. The *BRAF V600E* mutation is found in 8–10% of metastatic CRC cases and confers a worse prognosis [29]. In contrast to the melanoma setting, single-agent BRAFV600E inhibitors showed very limited activity in patients with advanced CRC. Similarly to the *KRAS G12C*-mutated CRC setting, BRAFV600E inhibition, in this CRC tumor context, showed an immediate upregulation of EGFR, this finding leading to the development of dual-blockade therapeutic strategies with the addition of an EGFR-directed monoclonal antibody [30,31]. Clinical phase Ib studies, with the combination of encorafenib with cetuximab and dabrafenib with panitumumab, have shown enhanced activity in this *BRAF* V600E-mutated CRC setting. Moreover, studies in preclinical models have shown that combined BRAF and MEK inhibition achieve a more thorough abrogation of ERK signaling, thereby forestalling the development of acquired resistance and suppressing the paradoxical activation of the ERK pathway. A BRAF-MEK dual blockade using dabrafenib–trametinib has shown enhanced activity in metastatic *BRAF* V600E-mutated CRC [32]. In the BEACON phase III trial, the triple combination of encorafenib, binimetinib, and cetuximab, as well as the encorafenib and cetuximab doublet, demonstrated an improved ORR, PFS, and OS compared to FOLFIRI or irinotecan with cetuximab [33], leading to the approval of encorafenib–cetuximab for previously treated *BRAF* V600E metastatic CRC. Another randomized phase II study demonstrated the activity of a combination of vemurafenib, irinotecan, and cetuximab in this setting [34]. The triple inhibition of BRAF, MEK, and EGFR was explored in the phase II single-arm ANCHOR study in 95 previously untreated patients, reporting an ORR of 47%, with a median PFS of 5.8 months and a median OS of 18.3 months [35]. Currently recruiting, the BREAKWATER phase III trial is studying the doublet with or without chemotherapy versus standard-of-care in a first-line setting (NCT04607421).

#### 2.1.5. PI3K-AKT-mTOR

Proliferation and survival are also maintained by the PI3K-AKT-mTOR axis, which promotes epithelial–mesenchymal transition and affects cell migration, leading to the emergence of metastases. Hyperactivation of this pathway can be caused by different mechanisms, such as the activation of RTKs, mutation or amplification of *AKT* or *PI3CA*, or a reduced expression or inactivation of PTEN, an AKT inhibitor [36].

Inavolisib is an oral PI3K inhibitor that prevents the activation of this signaling pathway in PI3K-overexpressing tumor cells. It binds to the ATP-binding site of PI3Kα, interfering with the phosphorylation of PIP2 to PIP3. The phase I/Ib study INTRINSIC (NCT04929223) is currently evaluating the safety and efficacy of inavolisib in combination with cetuximab or bevacizumab, in addition to other treatment approaches that include atezolizumab (an anti-programmed death-ligand 1 [PD-L1]) and tiragolumab (anti-TIGIT), alone or with bevacizumab, and divarasib combined with chemotherapy and cetuximab.

### 2.2. Promoting Sustained Angiogenesis

**Figure 3:**
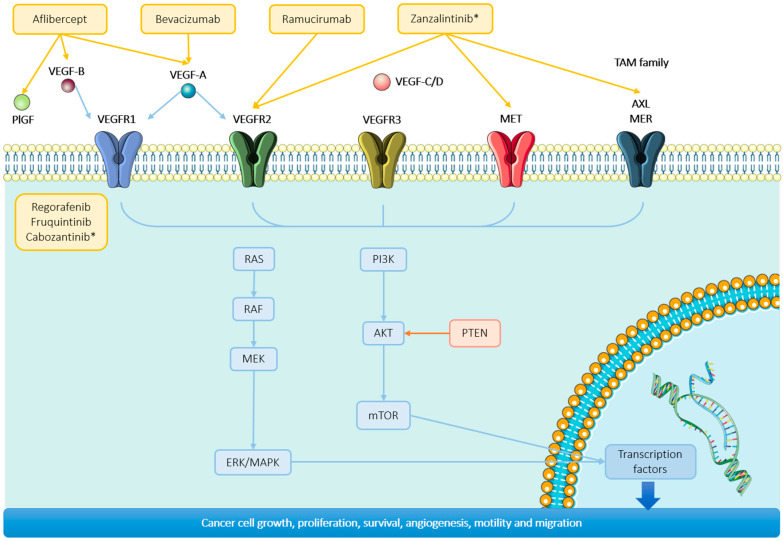
Targeted therapies in the vascular endothelial growth factor (VEGF) pathway. Approved treatments and treatments under investigation in VEGF signaling pathway. Treatments in ongoing clinical trials are marked with an asterisk (not approved by the FDA).

The vascular endothelial growth factor (VEGF) receptor is an RTK that promotes angiogenesis, leading to the formation of abnormal and malfunctioning blood vessels, and it is overexpressed in 40–60% of colorectal tumors [37]. Anti-VEGF therapies are essential in the treatment armamentarium for metastatic CRC (Figure 3).

Bevacizumab, a VEGF-A monoclonal antibody, has shown benefits in first- and second-line settings when combined with chemotherapy [38]. Aflibercept, a VEGF-A and -B and PLGF inhibitor, and ramucirumab, a VEGF receptor 2 (VEGFR2) inhibitor, demonstrated benefit in PFS and OS in a second-line setting in the VELOUR and RAISE trials, respectively [39,40]. Moreover, recent investigations have reported a statistically significant benefit in the third-line setting for bevacizumab combined with trifluridine/tipiracil in the SUNLIGHT trial, with a benefit in PFS (5.6 vs. 2.4 months) and OS (10.8 vs. 7.5 months) for the combination [41]. Another third-line option is regorafenib, an oral multikinase inhibitor (VEGFR1–3, FGFR1, PDGFR, c-KIT, RET, and TIE2) with antiangiogenic effects, which showed a better DCR (41% vs. 14%) and OS (6.4 vs. 5 months) versus placebo in the CORRECT trial [42].

Recently, fruquintinib, a novel anti-VEGFR that targets VEGFR1, VEGFR2, and VEGFR3, demonstrated clinical benefits in heavily pretreated metastatic CRC patients in the FRESCO-2 trial, with a median OS of 7.4 months versus 4.8 months in the placebo group [43].

Further to this, it has been observed that antiangiogenic therapy enhances the efficacy of immunotherapy by different mechanisms. VEGF cannot only promote angiogenesis, but also plays an important immunosuppressive role. In the tumor microenvironment (TME), VEGF interferes in the maturation of dendritic cells, repolarizes tumor-associated macrophages to M2-like phenotypes, and accumulates regulatory T cells. It also activates TOX, a transcription factor in CD8+ T cells, initiating their conversion to an ‘exhausted’ state, and upregulates different checkpoint inhibitor receptors such as PD-L1. Moreover, abnormal vessels created in the TME are an obstacle for the arrival of immune cells. They also perpetuate hypoxia within the tumor, which is itself an enhancer of angiogenesis, and concurrently recruit immunosuppressive immune cells by secreting chemotactic factors [44]. Considering this intricate relationship, combinations of anti-VEGF therapies and immune checkpoint inhibitors (ICIs) have been studied in different tumor types.

Cabozantinib, a multi-target TKI, including VEGFR2 and MET, with immunomodulatory properties, was combined with durvalumab in the CAMILLA phase I/II trial in different gastrointestinal tumors, including microsatellite stable (MSS) CRC, which is associated with a poorer response to immunotherapy (see below, under “Avoiding Immune Destruction”). The CRC cohort included 31 patients who had received two or more prior lines of therapy, achieving an ORR of 28% and a DCR of 86%, with a median PFS of 3.7 months and median OS of 9.1 months [45]. However other clinical trials combining a TKI with an ICI failed to demonstrate efficacy over standard-of-care in MSS CRC patients, including lenvatinib plus pembrolizumab in LEAP-017 [46], and cobimetinib (a MEK inhibitor) plus atezolizumab in the Imblaze370 phase III trial [47].

Zanzalintinib (XL092) is a novel multikinase inhibitor that targets MET, VEGFR2, AXL, and MER, impacting tumor growth and angiogenesis. In preclinical studies, zanzalintinib has shown activity in combination with anti-PD-1 antibodies. A phase Ib dose escalation study of zanzalintinib alone or in combination with atezolizumab reported a manageable safety profile in patients with solid tumors, including CRC [48]. The ongoing phase III STELLAR-303 is comparing zanzalintinib plus atezolizumab versus regorafenib in patients with metastatic CRC without hepatic involvement who have progressed on standard-of-care therapies, and who have not received regorafenib, trifluridine/tipiracil, or immunotherapy (NCT05425940).

### 2.3. Avoiding Immune Destruction

**Figure 4:**
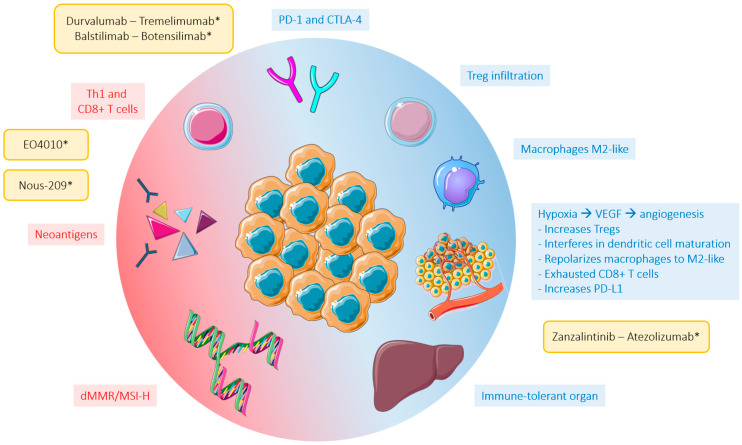
Down-regulators and up-regulators of immunity in tumor microenvironment. Down-regulators (blue) and up-regulators (red) of immunity in tumor microenvironment. Treatments in ongoing clinical trials are marked with an asterisk (not approved by the FDA). Abbreviations: PD-1, programmed cell death protein 1; CTLA-4, cytotoxic T-lymphocyte antigen 4; dMMR, deficient mismatch repair; MSI-H, microsatellite instability-high; VEGF, vascular endothelial growth factor; Treg, regulatory T cells.

The immune system is designed to defend and protect the body against infections and its own diseased cells. This intricate network of molecules and specialized cells has the ability to recognize foreign structures and eliminate possible threats. However, cancer cells are adept at evading immune destruction by creating an immunosuppressive environment. Several upregulators and downregulators of immunity have been described (Figure 4). Some proposed downregulatory mechanisms in the TME, in addition to the above-mentioned mechanisms associated with VEGF, are the secretion of transforming growth factor beta (TGF-β) and the recruitment of immunosuppressive cells, such as regulatory T cells [6].

Immunotherapy is a broad term that includes different treatment strategies to enhance immunity to fight cancer cells. In a similar manner to the passive and active immunity achieved from antibody therapy and vaccination, respectively, immunotherapy has been classified as “passive”, directly using immune effectors such as monoclonal antibodies or ADCs, and “active”, modulating regulatory mechanisms to activate the immune system, such as antigen vaccines or anti-cytotoxic T-lymphocyte antigen 4 (CTLA-4) monoclonal antibodies [49]. However, these strategies are not individually selected on the basis of their deficiency or absence in each patient, but rather because they are known to activate the immune response in general. This leads to a series of immune-related side effects that have limited the use of these therapies. One of the most studied immune evasion mechanisms is the programmed cell death pathway [49].

Programmed cell death protein 1 (PD-1) limits the activity of T cells in peripheral tissues by binding them to its ligands, PD-L1 (also known as B7-H1 and CD274) or PD-L2 (also known as B7-DC and CD273), and inhibiting cytokine production, as well as proliferation and survival signals [50]. Not only is PD-1 highly expressed in tumor-infiltrating lymphocytes (TILs), but PD-L1 is also upregulated in tumor cells in many different cancer types, which has led to the design of anti-PD-1 therapies to overcome this immunosuppressed environment [51]. Consequently, tumor PD-L1 overexpression could be expected to correlate with an anti-PD-1 therapy response. Despite some good examples, such as for NSCLC with patients selected by their high expression of PD-L1 [52], this ideal situation does not apply to every tumor type [53]. Thus, PD-L1 expression represents a significant but not an absolute predictive biomarker of response to anti-PD-1 therapies. In the case of CRC, anti-PD-1 response has been associated with MSI or dMMR tumors, and not in mismatch repair-proficient or MSS tumors [54,55]. Therefore, MSI status is currently the main biomarker for immunotherapy response in advanced CRC; dMMR and MSI have been ascribed to the loss of function of any of the DNA mismatch machinery proteins, including MSH2, MSH6, MLH1, and PMS2, which leads to an abundance of DNA replication errors and, hence, a hypermutated phenotype. These tumors normally present with a high tumor mutational burden (TMB) and, by consequence, an inflammatory TME, which comprises TILs, memory cells, and cytotoxic T lymphocytes (CTLs) [56].

Pembrolizumab, a PD-1 inhibitor, has been approved by the Food and Drug Administration (FDA) and the European Medicines Agency (EMA) as a first-line treatment for dMMR/MSI metastatic CRC based on the pivotal phase III KEYNOTE-177 trial [57]. Pembrolizumab showed an improvement in PFS and fewer treatment-related adverse events compared to standard-of-care. However, no significant differences were observed in OS, probably related to the cross-over design of the study. Additionally, the combination of nivolumab and ipilimumab, PD-1 and CTLA4 inhibitors respectively, has been approved as a second-line therapeutic option for dMMR/MSI tumors [58]. Furthermore, the CheckMate-8HW phase III trial (NCT04008030) in patients with unresectable or metastatic CRC with dMMR/MSI-high (MSI-H), comparing first-line treatment with nivolumab plus ipilimumab versus standard-of-care, recently reported that a median PFS was not reached in 171 patients who received the immunotherapy doublet and was 5.8 months among 84 patients who received the standard therapy after a median follow-up of 24.3 months. The combination of ipilimumab and nivolumab entailed a 79% reduction in the risk of disease progression or death (hazard ratio 0.21, 95% CI [0.14, 0.32]; *p* < 0.0001) [59]. Further data on secondary endpoints, including OS, are pending.

Despite positive results, about 15–30% of dMMR/MSI CRC do not respond to immunotherapy, and about 20–25% of patients are refractory, with an initial benefit and late progression, suggesting that current patient selection criteria are not ideal. Some clinical, biological, and molecular features have been associated with an inferior benefit from ICIs, such as the presence of liver metastases [60,61,62] or angiogenesis, as mentioned earlier.

The liver is an immune-tolerant organ, secondary to its considerable vascularization and flow of antigens, received principally from the gastrointestinal tract, with its immunosuppressive environment preventing it from continuous immune reactions to these circulating antigens. In this context, it is coherent that hepatic metastases can be less responsive to immunotherapy. Further to this, it has been observed that not only do the hepatic lesions not respond, but neither does the systemic disease, leading to the hypothesis that liver lesions create a systemic suppression of immunity. Yu et al. [63] conducted a study on mice, injecting immunotherapy-sensitive cancer cells and creating two different models, with liver metastases or with lung metastases. The authors found that in the absence of hepatic metastases, tumors had abundant CD8+ T cells, while tumors with liver disease had fewer tumor-specific CD8+ T cells. Additionally, they treated liver disease with radiotherapy, anti-PD-L1, or a combination of both, observing an increased presence of interferon gamma production by CD4+ and CD8+ T cells in tumors treated with radiotherapy, hypothesizing that it could restore the efficacy of ICIs in this scenario. In another study, Lee et al. associated regulatory T cells and intratumoral CD11b+ monocytes with this immunosuppressive environment in in vivo tumor models of liver metastases in immunocompetent mice, suggesting that combining anti-CTLA4 or EZH2 inhibitors with anti-PD-1 could improve clinical results [64]. This has led to the exclusion of patients with hepatic metastases in some trials exploring combinations with immunotherapy, such as the previously mentioned STELLAR-303 trial.

In addition to PD-L1 and MSI status, TMB, a measure of mutations per megabase (mutations/Mb) in cancer cells, has been considered predictive of the response to immunotherapy, on the basis that tumors with a high TMB present a higher amount of neoantigens and have more susceptibility to creating an immune response. However, the utility of this biomarker is yet to be defined, and it is unlikely to be homogenous for every tumor type. Rousseau et al. evaluated 137 patients with advanced CRC treated with ICIs and observed a longer median OS in patients with TMB >10 mutations/Mb compared to those with <10 mutations/Mb. Nonetheless, this difference disappeared when stratified by dMMR status or pathogenic mutations in polymerase epsilon or D1. Moreover, only patients with mismatch repair-proficient head and neck cancer, NSCLC, or melanoma showed a better OS with a higher TMB [65]. It has also been suggested that the quantity of mutations may not be as important as their quality. For example, mutations influencing RNA splicing can generate more neoantigens in comparison with other types of mutations, although each and every one is included in the TMB measurement [66]. There are many ongoing clinical trials investigating potential combination therapies, including immunotherapy in MSS as well as MSI CRC, to improve patient outcomes.

Regarding MSI disease and its association with *BRAF* mutations, the SEAMARK phase II trial is comparing a combination of encorafenib and cetuximab plus pembrolizumab, with pembrolizumab in monotherapy in untreated *BRAF* V600E-mutated MSI CRC patients (NCT05217446). New strategies for MSI include Nous-209, a vaccine encoding 209 neoantigens that are shared across sporadic and hereditary MSI tumors. It has been tested in monotherapy in a phase Ib study in Lynch syndrome patients, with no serious adverse events [67]. Currently, this new vaccine is being studied in the NOUS-209–01 clinical trial, in combination with pembrolizumab in patients with unresectable or metastatic dMMR or MSI-H CRC, gastric, or gastroesophageal junction tumors (NCT04041310).

As for MSS CRC, and with a similar approach, the AUDREY study (NCT05589597) is a multicenter phase I/II trial assessing the safety, tolerability, immunogenicity, and preliminary efficacy of EO4010, containing five synthetic peptides presenting molecular mimicry with tumor-associated antigens highly expressed in CRC, as a monotherapy and in combination with nivolumab for treatment of advanced CRC, regardless of MSI status. Additionally, combinations of both anti-PD1 and anti-CTLA4 have shown better responses than ICIs in monotherapy. Botensilimab (anti-CTLA4) and balstilimab (anti-PD-1) have been studied in a phase Ia/b trial that included previously treated MSS CRC patients, with an ORR of 22%, a DCR of 73%, and the median duration of response not reached. The 12-month OS rate was 61%. Interestingly, of the 13 patients with an objective response, none had a TMB of ≥10 mutations/Mb, and only one was PD-L1-positive (≥1% combined positive score) [68]. A randomized phase II trial is evaluating botensilimab alone or in combination with balstilimab in comparison with standard-of-care (regorafenib or trifluridine/tipiracil) in refractory metastatic CRC (NCT05608044). For combinations with antiangiogenics, refer to “Promoting Sustained Angiogenesis”.

### 2.4. Genome Instability and Mutation

As described by Hanahan and Weinberg, cancer cells gain certain abilities through their ability to mutate [6]. By increasing sensitivity to mutagenic agents and defective maintenance machinery, mutation rates are much higher than in normal cells, providing a variety of clones that can prevail in the tumor environment.

It is of utmost importance to maintain the integrity of a cell’s DNA and for the cell to monitor for any errors that may put at risk the correct transmission of information and potentially the viability of the cell. Different DNA repair mechanisms exist in all cells to address this [69]. Firstly, direct reversal of the chemical reaction that caused the DNA damage, which is important in repairing UV light damage. Secondly, the removal of damaged bases, known as excision repair, which can be accomplished by removing the damaged base (base-excision repair, BER), the removal of an oligonucleotide containing the damage (nucleotide-excision repair, NER), or the scanning of the DNA during replication by the DNA polymerase (MMR). And lastly, after completion of replication, if previous mechanisms have failed, double-strand DNA can be repaired by homology-directed repair (HR) and nonhomologous end joining (NHEJ). Most cancer cells have defects in one of these repair systems, including dMMR in CRC.

Poly (ADP-ribose) polymerase (PARP) not only has an important role in BER, but also helps recruit other repair proteins that play a part in HR and regulates the expression of BRCA1 and RAD51. The inhibition of PARP in a cell with an existing defect in the DNA damage repair system leads to synthetic lethality, a situation in which the disruption of two genes simultaneously is not viable, resulting in cell death [69]. This is the rationale for the use of PARP inhibitors in tumors with mutated *BRCA1*/2. Moreover, this synthetic lethality is associated with the release of neoantigens, resulting in an increased immune response that leads to the idea of combining PARP inhibitors and ICIs. Other effects of PARP inhibitors have been linked to enhanced immunity and have laid the groundwork for studies of combination strategies. PARP inhibitors can upregulate the expression of PD-L1 [70,71] and also play a role in activating CD8+ and CD4+ T cells, as well as blocking macrophage differentiation towards an M2 subtype, creating a more inflammatory TME [72]. In line with this, the phase II PEMBROLA trial is recruiting patients with HR-deficient metastatic CRC, defined by the presence of a *BRCA* mutation or a low RAD51 score (<10%), to receive pembrolizumab and olaparib (PARP inhibitor). A required inclusion criteria is oxaliplatin sensitivity, defined as a PFS of at least 9 months in the first-line setting, having received at least 8 cycles of FOLFOX or 6 cycles of XELOX (capecitabine and oxaliplatin).

While CRC harboring dMMR or MSI is highly sensitive to ICIs, the majority of CRC patients are classified as MSS and therefore have a poor response to immunotherapy. In this context, PARP inhibitors could potentially create synergism in combination with ICIs. In the DAPPER phase II trial (NCT03851614), patients with MSS CRC, pancreatic adenocarcinoma, or leiomyosarcoma were randomized to receive a combination of durvalumab (anti PD-1) plus olaparib or cediranib (VEGF1-3 inhibitor). Preliminary data show that the durvalumab plus olaparib combination increased immune cell infiltration and benefits in some leiomyosarcoma patients [73], with further data pending.

DNA damage is also key in the regulation of the cell cycle. Cyclin-dependent kinases (CDK) regulate different steps in the cycle in partnership with several cyclins, forming a cyclin–CDK complex. The G2/M cell cycle checkpoint prevents a cell entering mitosis if DNA damage is present, and it is driven by cyclinB-CDK1. When DNA damage occurs, the ATR-CHK1-WEE1 pathway is activated and WEE1 maintains CDK1 phosphorylated in its inactive form, preventing progression to the M phase and allowing for DNA damage repair. In preclinical studies, the heterozygous deletion of *WEE1* resulted in genomic instability and, ultimately, in tumor development in a murine mammary epithelium [74]. Adavosertib (AZD-1775) is the first highly potent and selective WEE1 inhibitor. In preclinical studies, it has demonstrated increased cytotoxicity in combination with 5-fluorouracil in p53-mutated CRC [75]. It was evaluated in the FOCUS4-C phase II trial in patients with *TP53/RAS-*mutant metastatic CRC after the completion of standard chemotherapy, compared to active monitoring. This study met its primary endpoint, with adavosertib improving the PFS (3.6 vs. 1.9 months), although it did not show an OS benefit [76]. A phase Ib study has been designed to determine if a combination of adavosertib and irinotecan is safe and effective in *RAS-* or *BRAF*-mutated CRC in a second-line setting (NCT02906059). Azenosertib (ZN-c3) is another potent WEE1 inhibitor that has proven to be safe in phase I trials in platinum-resistant ovarian cancer and advanced uterine serous carcinoma patients and is currently being studied in *BRAF-*mutated CRC, in combination with encorafenib and cetuximab (NCT05743036).

### 2.5. Reprogramming Cellular Metabolism

Cancer cells change their glucose metabolism into what is known as “aerobic glycolysis” [6]. Normal cells produce energy through glycolysis in the cytoplasm, the Krebs cycle, and oxidative phosphorylation in the mitochondria. Under anaerobic conditions, glycolysis is favored but pyruvate is not directed to the mitochondria but transformed into lactate. The efficiency of aerobic metabolism produces 38 adenosine triphosphate (ATP) molecules for every glucose molecule, whereas only 2 ATP molecules result from anaerobic glycolysis. Contrary to the general belief, cancer cells transform their metabolism into the latter, less efficient pathway. This pathway is faster, and can be upregulated by increasing glucose transporters, such as GLUT1, raising glucose import into the cytoplasm. This concept led to the use of ^18^F-fluorodeoxyglucose (FDG) as a contrast for positron emission tomography [77]. Moreover, hypoxic conditions, present in many tumors, also contribute to enhancing glucose transporters and other enzymes of the glycolytic pathway through the upregulation of hypoxia-inducible factor-1α (HIF1α) and HIF2α transcription factors [6]. Different targets arise in this sphere, such as HIF2α and isocitrate dehydrogenase 1–2 (IDH). Belzutifan is an HIF2α inhibitor with FDA approval in renal cell carcinoma and von Hippel–Lindau-associated tumors [78]. Ivosidenib is an IDH1 inhibitor that has been approved for acute myeloid leukemia [79] and cholangiocarcinomas [80] and is being investigated in *IDH*-mutated gliomas [81], among other tumors.

**Figure 5:**
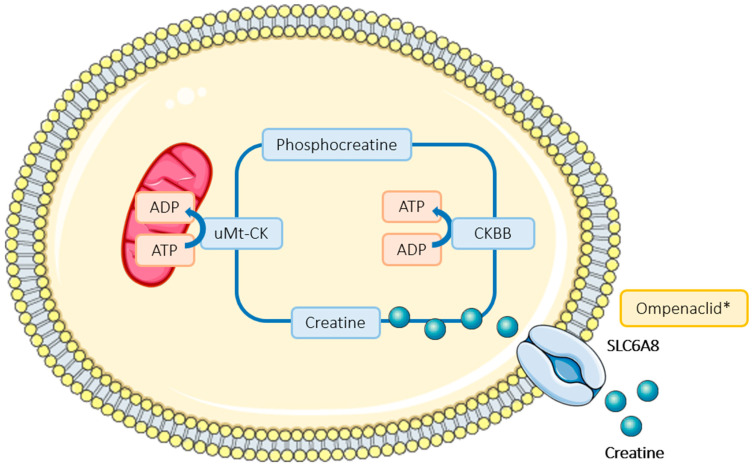
Inhibiting creatine kinase circuit in CRC cells. Creatine can be produced endogenously or taken up from the circulation via the creatine transporter (SLC6A8). Creatine is phosphorylated to form phosphocreatine, using adenosine triphosphate (ATP) in a reaction catalyzed by ubiquitous mitochondrial creatine kinase (uMt-CK). When energy is required, cytosolic isoforms of creatine kinase (CKBB) hydrolyze phosphocreatine, thus regenerating ATP and creatine. Treatments in ongoing clinical trials are marked with an asterisk (not approved by the FDA). Abbreviations: ADP, adenosine diphosphate.

In CRC, creatine kinase-B promotes tumor growth and survival under hypoxia, as it generates ATP by catalyzing phosphocreatine, which is imported into the cell through the SLC6A8 transporter. Ompenaclid (RGX-202–01) is an oral inhibitor of the SLC6A8 transporter that induces apoptosis by inhibiting creatine importation into the cell, therefore reducing ATP levels (Figure 5). It has been suggested that *RAS-*mutant cancer cells could be more sensitive to ATP depletion because of their increased metabolism. However, this new molecule has shown a suppression of growth in patient-derived xenografts for both *KRAS* wild-type and *KRAS-*mutant tumors [82]. A phase Ia clinical trial in refractory *KRAS-*mutant CRC has been completed, showing antitumor activity without dose-limiting toxicity. The phase Ib part, evaluating ompenaclid in combination with FOLFIRI-bevacizumab in the second-line setting, showed interesting results, with a response rate of 56%, reflecting better-than-expected results compared to standard-of-care [83]. The ongoing phase II RGX-202-002 trial is comparing second-line FOLFIRI-bevacizumab in combination with ompenaclid versus placebo in patients with *KRAS-*mutated advanced CRC (NCT05983367).

### 2.6. Avoiding Cell Death

Programmed cell death or apoptosis is a natural mechanism that occurs during development and aging in all healthy tissues. It has both a homeostatic function, as it controls the cell population, and a defensive function, as it eliminates damaged or diseased cells. Activating pathways of apoptosis include the extrinsic pathway, the intrinsic pathway, and the perforin/granzyme pathway. All three converge in the same execution pathway, initiated by caspase-3, which leads to DNA fragmentation, degradation of the cytoskeleton, and the formation of apoptotic bodies. Nearby macrophages and parenchymal cells phagocyte the apoptotic bodies, such that little or no inflammatory reaction will result from the process of apoptosis, in contrast to necrosis [84].

Cancer cells not only present with the ability to sustain proliferative signaling, as mentioned earlier, but also suppress apoptosis to resist cell death. By upregulating anti-apoptotic proteins, such as Bcl-2, or by downregulating pro-apoptotic proteins, such as Bax, tumor cells gain the capacity to avoid programmed cell death [6]. One of the most mutated genes present in tumors with a critical role in the regulation of this process is *TP53*. This tumor suppressor gene produces the p53 protein and is known as the guardian of the genome, as it acts as a transcription factor involved in DNA repair, cell cycle control, autophagy, and apoptosis [85]. Different strategies to target p53 have been attempted across many cancer types, including targeting mutated p53 directly to restore its function, inducing its degradation or depletion, and inducing cell death through synthetic lethality, such as with Wee1 inhibitors [86]. As for the first approach, PRIMA-1^met^ (APR-246) is a methylated analog of PRIMA-1, a p53 reactivator, which has been tested in CRC cells. This compound induces apoptosis in *p53*-mutated CRC cells but has also been shown to limit proliferation, regardless of *p53* status [87]. To our knowledge, no clinical trials have been published testing the use of PRIMA-1^met^ in CRC.

Other known targets in the programmed cell death circuit are found in the extrinsic pathway, involving transmembrane receptors called death receptors (DR) from the tumor necrosis factor (TNF) receptor superfamily. One of these receptors, DR5, is being studied as a target in CRC. Aplitabart (IGM-8444) is an IgM antibody that has been designed to bind to DR5 in cancer cells to induce apoptosis [88]. The IGM-8444-001 clinical trial is a first-in-human phase Ia/b study to test the safety and tolerability of aplitabart in patients with different tumor types (NCT04553692). In patients with CRC, the drug is being tested in combination with FOLFIRI, with or without bevacizumab, in the second line.

Napabucasin is an oral treatment that induces apoptosis, by activating caspase 3/7/8, and cell cycle arrest, and suppresses proliferation, invasion, and metastasis in cancer cells. More importantly, it has the ability to suppress cancer stem cell proliferation and inhibit the expression of stemness-associated genes, including STAT3 [89]. Despite the observed effects in vitro, napabucasin failed to improve OS in combination with FOLFIRI, compared to FOLFIRI alone, in previously treated CRC patients in the CanStem303C phase III trial [90].

## 3. Conclusions

**Figure 6:**
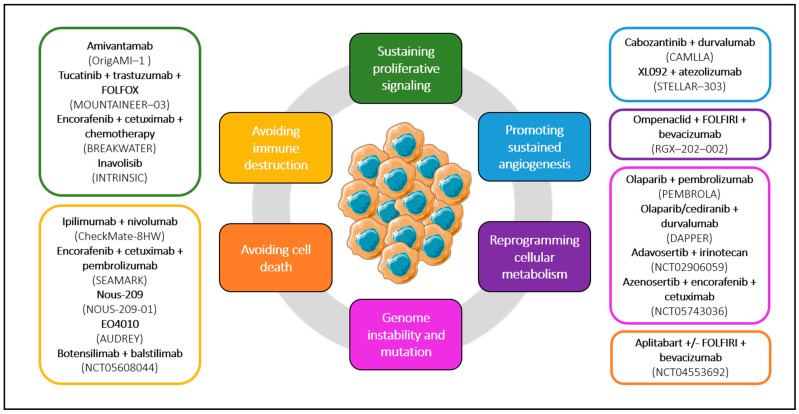
New treatment strategies in CRC in the context of the hallmarks of cancer.

The cornerstone of the current management of metastatic CRC includes chemotherapy and biomarker-driven therapies, notably anti-EGFR, anti-VEGF, and anti-HER2 agents, KRAS G12C inhibitors, and BRAF and MEK inhibitors, as well as ICIs and combinations thereof. New treatment strategies are being investigated in CRC with our increasing understanding of tumor biology and novel molecular targets (Figure 6). The hallmarks of cancer are a reflection of the paramount progress achieved in the past 20 years, and they have served as a guide to decipher the multiple currently available treatment options that are being investigated in CRC. The unmasked targets in CRC cells help sustain proliferative signaling, induce angiogenesis, resist apoptosis, adapt cellular metabolism, and avoid immune destruction, with various targeted therapies being studied in clinical trials giving promising results. New perspectives are being integrated into our understanding of cancer cells, recognizing them as part of a systemic disease that transcends the boundaries of their microenvironment [91]. Environmental elements such as diet, physical activity, microbiome, stress, aging, and circadian rhythms are not only considered risk factors for developing cancer but also play crucial roles in the progression of the disease. Consequently, these factors could potentially serve as targets for new therapeutic strategies. The further understanding of this complex network must continue, as it is paramount for developing novel treatment options and positively impacting patient outcomes and quality of life.
